# Impact of initial temporary abdominal closure in damage control surgery: a retrospective analysis

**DOI:** 10.1186/s13017-018-0204-3

**Published:** 2018-09-15

**Authors:** Parker Hu, Rindi Uhlich, Frank Gleason, Jeffrey Kerby, Patrick Bosarge

**Affiliations:** 1 Division of Acute Care Surgery, Department of Surgery, University of Alabama at Birmingham, Birmingham, AL USA; 2 Division of Acute Care Surgery, Department of Surgery, University of Alabama at Birmingham, 701 19th Street South, 112 Lyons-Harrison Research Building, Birmingham, AL 35294 USA; 3 Department of Surgery, University of Alabama at Birmingham, Birmingham, AL USA

**Keywords:** Damage control surgery, Temporary abdominal closure, Loss of domain, ABThera, Bogota bag, Vacuum packing

## Abstract

**Background:**

Damage control surgery has revolutionized trauma surgery. Use of damage control surgery allows for resuscitation and reversal of coagulopathy at the risk of loss of abdominal domain and intra-abdominal complications. Temporary abdominal closure is possible with multiple techniques, the choice of which may affect ability to achieve primary fascial closure and further complication.

**Methods:**

A retrospective analysis of all trauma patients requiring damage control laparotomy upon admission to an ACS-verified level one trauma center from 2011 to 2016 was performed. Demographic and clinical data including ability and time to attain primary fascial closure, as well as complication rates, were recorded. The primary outcome measure was ability to achieve primary fascial closure during initial hospitalization.

**Results:**

Two hundred and thirty-nine patients met criteria for inclusion. Primary skin closure (57.7%), ABThera™ VAC system (ABT) (15.1%), Bogota bag (BB) (25.1%), or a modified Barker’s vacuum-packing (BVP) (2.1%) were used in the initial laparotomy. Patients receiving skin-only closure had significantly higher rates of primary fascial closure and lower hospital mortality, but also significantly lower mean lactate, base deficit, and requirement for massive transfusion. Between ABT or BB, use of ABT was associated with increased rates of fascial closure. Multivariate regression revealed primary skin closure to be significantly associated with primary fascial closure while BB was associated with failure to achieve fascial closure.

**Conclusions:**

Primary skin closure is a viable option in the initial management of the open abdomen, although these patients demonstrated less injury burden in our study. Use of vacuum-assisted dressings continues to be the preferred method for temporary abdominal closure in damage control surgery for trauma.

## Background

Injury claims the lives of approximately 200,000 individuals in the USA annually, with hemorrhage and subsequent coagulopathy serving as the leading causes of preventable death [1–4]. Many surgical advancements have been achieved in recent times, but the development of damage control surgery (DCS) has revolutionized trauma care and led to a drastic reduction in mortality related to hemorrhagic shock. First named in the literature by Rotondo in 1993, DCS has proven to be the most effective means of limiting ongoing hemorrhage and reducing traumatic coagulopathy utilized today [5]. The employment of DCS and temporary abdominal closure (TAC) techniques began to staunchly reduce the mortality attributed to hemorrhagic shock, though with this decline came the increased recognition of complications associated with the open abdomen.

The initial techniques described for TAC consisted of primary skin closure (PSC) with either suture or towel clips [6, 7], followed by improvised plastic silos or sterilized IV bags sewn to the skin, named by Mattox as Bogotá bags (BB) [8]. While these measures were simple and cost effective, both focused simply on the containment of viscera and abdominal packings without providing a means for effective drainage of intraperitoneal fluid or visceral expansion caused by ongoing resuscitation. As a result, patients demonstrated elevated rates of abdominal compartment syndrome [6, 9]. The concept of negative pressure wound therapy was incorporated to address these issues. Known as Barker’s vacuum-packing (BVP), these dressings have now become widely accepted and many modifications have since been described [10, 11]. At its core, BVP consists of a perforated, plastic sheet placed over the viscera that is then covered by either towels or GranuFoam sponge, before an occlusive dressing and negative pressure device are applied. The success of these rudimentary vacuum dressings inspired development of commercial products, such as the ABThera™ VAC system (ABT; KCI USA, San Antonia, TX). Improved outcomes with the ABT over BVP have been reported, with one prospective observational study reporting significant increases in overall rates of primary fascial closure (PFC) and 30-day mortality in a mixed surgical cohort. However, this observation did not persist in the trauma subpopulation [12].

Even with ongoing innovation, inability to re-approximate the fascial edges in the midline continues to be among the most feared complications of TAC. Failure to achieve PFC results in a large ventral hernia and loss of abdominal domain (LOD). The causes of LOD are multifactorial, but are primarily attributed to fascial retraction and increased intraabdominal pressure. PSC and BB do not prevent fascial retraction or adhesion of the bowel to the abdominal wall and have historically been associated with an increased risk of LOD and complications associated with an open abdominal wound [13]. Vacuum-mediated closure methods have generally been regarded as superior due to their ability to drain peritoneal effluent while also providing continuous fascial traction toward the midline. As a result, many current management algorithms advocate for the use of some form of negative pressure wound therapy for initial management of the open abdomen [14–16].

The initial method for TAC may influence rates of LOD and clinical outcomes [17–20]. Success with primary fascial approximation during the initial hospitalization varies widely in the literature, with rates of PFC of 29–100% reported for vacuum-assisted dressings [6, 11, 21–24] compared to 40–75% for PSC [6, 25] and 12–82% for BB [6, 26–28]. Widespread acceptance of vacuum dressings, in particular commercial devices such as ABT, as the standard of care for TAC has led to cheaper and more readily available methods such as BB and PSC to be abandoned despite few comparative studies existing to appropriately guide therapy [14]. Additionally, most available data on the subject predates the era of damage control resuscitation (DCR), which may limit visceral edema and potentially negate previous complications experienced with TAC [29]. We sought to evaluate the role of initial TAC on eventual PFC and prevention of LOD.

## Methods

We performed a retrospective review of all trauma patients admitted to the University of Alabama at Birmingham Medical Center (UABMC) from 2011 to 2016. UABMC is an American College of Surgeons (ACS)-verified level 1 trauma center that serves as a tertiary referral center for the state of Alabama, with approximately 3500 trauma admissions per year. A registry of all trauma patients containing demographics, injuries, and injury severity is maintained by the trauma service.

All patients ≥ 18 years old admitted to the trauma service undergoing exploratory laparotomy at the time of admission were eligible for inclusion. Those patients undergoing DCS, defined as laparotomy with TAC following injury, were included for analysis. Patients receiving PFC at their initial operation or suffering a traumatic hernia preventing eventual PFC were excluded. The primary outcome of interest was the ability to achieve PFC based on initial closure technique during the index hospitalization. Secondary outcomes of interest were complications related to DCS and an open abdominal wound (fistula, ongoing bleeding, fascial dehiscence, abdominal abscess) as well as hospital mortality. PFC was defined as primary approximation of the fascia with suture repair. Fistula and dehiscence were identified clinically, with fistula defined as persistent communication between abdominal viscera and either the atmosphere or through the abdominal wall. Dehiscence was defined as any clinically apparent disruption of fascial closure. Ongoing significant bleeding was defined by bleeding requiring unplanned abdominal re-exploration. Abscess was identified intraoperatively or following percutaneous drainage with positive culture results.

Demographic and operative data were obtained from the electronic medical record. Operative reports were reviewed to determine the method of TAC. Four different types of abdominal closure were identified at the initial operations during the defined study period: PSC (all with running, monofilament suture); ABT, an improvised non-occlusive vacuum dressing using a modified BVP with GranuFoam rather than the standard towel; or a BB fashioned with a sterilized 3-L I.V. fluid bag. Patients were stratified into cohorts by the type of initial TAC for analysis.

Values were expressed as mean ± standard deviation or proportion (percentage). Categorical variables were compared using Pearson’s *χ*^2^ test, while continuous variables were compared using one-way ANOVA. Pairwise comparisons were performed post hoc with pairwise *χ*^2^ testing or by Tukey’s method in the event of significance. Multivariate logistic regression was used to determine the association of abdominal closure technique with PFC, adjusting for the preselected potentially confounding covariates of age, gender, mechanism of injury, injury severity score (ISS), and massive transfusion requirement (≥ 10 units pRBC/24 h). An a priori *p* value ≤ 0.05 was set to identify statistical significance. Similar adjusted regression analysis was further performed for hospital mortality and complications identified as significant on univariate analysis.

## Results

Two-hundred and thirty-nine patients were identified during the study period and included for analysis. Patients shared similar demographics among the cohorts (Table 1). Overall, patients were predominately male (82%) and were more likely to suffer penetrating injury (55.2%). PSC was the most commonly used method for TAC (57.7%), followed by BB (25.1%), ABT (15.1%), and then BVP (2.1%). Injury patterns among the different cohorts were similar except for an increased proportion of pancreatic wounds in the PSC vs ABT cohorts (*p* = 0.009).

**Table 1:** Comparison of demographic and clinical data by initial closure technique

	Overall (*n* = 239)	Skin Only (*n* = 138)	ABThera System (*n* = 36)	Bogotá Bag (*n* = 60)	Barker’s vacuum packing (*n* = 5)	*p* value
Demographics						
Age (years)	38.12 ± 14.70	37.85 ± 14.44	41.75 ± 15.62	36.26 ± 13.94	41.71 ± 22.95	0.32
Gender (%)						
*Male*	196 (82.0)	107 (77.5)	33 (91.7)	51 (85.0)	5 (100)	0.13
*Female*	43 (18.0)	31 (22.5)	3 (8.3)	9 (15.0)	0 (0)	
Body mass index (kg/m^2^)	29.73 ± 6.72	29.69 ± 6.71	30.20 ± 6.47	29.00 ± 6.65	35.86 ± 8.04	0.17
Mechanism of injury (%)						
*Blunt*	107 (44.8)	59 (42.8)	19 (52.8)	29 (48.3)	0 (0)	0.14
*Penetrating*	132 (55.2)	79 (57.2)	17 (47.2)	31 (51.7)	5 (100)	
Ethnicity (%)						
*Caucasian*	111 (46.4)	65 (47.1)	16 (44.4)	28 (46.7)	2 (40.0)	0.72
*African American*	121 (50.6)	70 (50.7)	18 (50.0)	30 (50.0)	3 (60.0)	
*Latin American*	6 (2.5)	3 (2.2)	1 (2.8)	2 (3.3)	0 (0)	
*Asian American*	1 (0.4)	0 (0)	1 (2.8)	0 (0)	0 (0)	
Injury						
Injury Pattern (%)						
*Major vascular*	29 (12.1)	10 (7.2)	7 (19.4)	12 (20.0)	0 (0)	0.09
*Pelvic*	21 (8.8)	9 (6.5)	7 (19.4)	5 (8.3)	0 (0)	0.09
*Splenic*	71 (29.7)	35 (25.4)	14 (38.9)	20 (33.3)	2 (40.0)	0.34
*Hepatic*	72 (30.1)	42 (30.4)	10 (27.8)	18 (30.0)	2 (40.0)	0.95
*Renal*	12 (5.0)	6 (4.3)	2 (5.6)	4 (6.7)	0 (0)	0.86
*Pancreatic*	21 (8.8)	6 (4.3)	6 (16.7)	7 (11.7)	2 (40.0)	0.005
*Gastric*	28 (11.7)	15 (10.9)	5 (13.9)	6 (10.0)	2 (40.0)	0.23
*Small bowel*	88 (36.8)	48 (34.8)	13 (36.1)	25 (41.7)	2 (40.0)	0.83
*Colorectal*	91 (38.1)	52 (37.7)	15 (41.7)	22 (36.7)	2 (40.0)	0.97
Clinical						
Injury Severity Score	25.68 ± 13.77	25.14 ± 14.20	29.53 ± 14.37	24.83 ± 12.42	22.80 ± 10.99	0.33
Admission lactate (mMol/L)	5.66 ± 3.887	4.79 ± 2.95	6.18 ± 3.49	7.27 ± 5.31	6.58 ± 3.26	< 0.001
Admission base excess (mMol/L)	−8.18 ± 5.69	−6.89 ± 4.95	−9.78 ± 5.46	−9.92 ± 6.65	−12.04 ± 5.76	< 0.001
Massive transfusion (%)	82 (34.3)	27 (19.6)	21 (58.3)	33 (55.0)	1 (20.0)	< 0.001
Units PRBC transfused first 24 h	9.27 ± 11.01	5.35 ± 4.98	13.78 ± 13.30	15.53 ± 15.35	9.80 ± 5.85	< 0.001
Units total blood products transfused first 24 h	19.49 ± 22.29	11.67 ± 11.44	29.17 ± 29.97	31.38 ± 28.48	22.80 ± 12.95	< 0.001

^*^Values displayed as mean ± SD unless otherwise specified. Estimates from *χ*^2^ and one-way ANOVA for categorical and continuous variables, respectively

Markers of injury severity were not significantly different among patients with ABT, BB, or BVP (Table 1). Patients, managed with PSC though, demonstrated significantly lower mean lactate than patients with BB closure (*p* < 0.001) and lower base excess (*p* < 0.001) than patients managed with either BB or ABT. Further, patients managed with PSC were significantly less likely to require massive transfusion (*p* < 0.001) and required significantly less average units of pRBCs (*p* < 0.001) or total blood products (*p* < 0.001) over the first 24 h compared with patients managed with BB or ABT.

Rates of PFC were highest among patients managed with PSC at initial operation, which was significantly higher than patients managed with BB (*p* = 0.001). Comparing patients managed with ABT versus BB, there were no significant differences in rates of PFC (94.4 vs 83.3%, *p* = 0.11). Among patients able to undergo PFC, rates of fascial dehiscence were lowest among patients with ABT or BVP, although not significant. There was no difference in hospital mortality between patients with initial ABT or BB closure (*p* = 0.88), although both were significantly elevated compared to PSC (*p* = 0.004). With regard to other hospital complications, the only significant difference among the cohorts was ongoing bleeding among patients with BB compared to PSC (*p* = 0.044) (Table 2).

**Table 2:** Comparison of clinical outcomes by initial closure technique

	Overall (*n* = 239)	Skin Only (*n* = 138)	ABThera System (*n* = 36)	Bogotá Bag (*n* = 60)	Barker’s vacuum packing (*n* = 5)	*p* value
Outcomes						
Length of stay (days)	28.70 ± 20.96	25.47 ± 16.94	35.67 ± 29.45	32.23 ± 22.38	25.20 ± 18.43	0.027
ICU length of stay (days)	22.04 ± 17.72	19.43 ± 15.07	28.58 ± 23.10	24.12 ± 18.80	18.80 ± 13.29	0.033
Number of abdominal operations	3.12 ± 1.77	2.75 ± 1.42	3.22 ± 1.48	3.80 ± 2.23	5.80 ± 8.01	0.001
Time to abdominal closure (days)	4.19 ± 4.25	3.35 ± 3.21	4.69 ± 3.89	5.70 ± 5.59	5.80 ± 8.01	0.002
Hospital mortality (%)	19 (7.9)	5 (3.6)	5 (13.9)	9 (15.0)	0 (0)	0.021
Achieve primary fascial closure	221 (92.5)	133 (96.4)	34 (94.4)	50 (83.3)	4 (80.0)	0.009
Loss of abdominal domain (%)	18 (7.5)	5 (3.6)	2 (5.6)	10 (16.7)	1 (20)	0.009
Fascial dehiscence	12 (5.0)	8 (5.8)	0 (0)	4 (6.7)	0 (0)	0.44
Repeat bleeding	19 (7.9)	7 (5.1)	2 (5.6)	8 (13.3)	2 (40.0)	0.011
Enterocutaneous fistula	12 (5.0)	4 (2.9)	2 (5.6)	6 (10.0)	0 (0)	0.19
Anastomotic leak	15 (6.3)	8 (5.8)	1 (2.8)	6 (10.0)	0 (0)	0.47
Early bowel obstruction	10 (4.2)	6 (4.3)	2 (5.6)	2 (3.3)	0 (0)	0.92
Intra-abdominal abscess	81 (33.9)	45 (32.6)	10 (27.8)	24 (40.0)	2 (40.0)	0.62
Wound infection	37 (15.5)	22 (15.9)	2 (5.6)	13 (21.7)	0 (0)	0.15

^*^Values displayed as mean ± SD unless otherwise specified. Estimates from *χ*^2^ and one-way ANOVA for categorical and continuous variables, respectively

On multivariate evaluation with logistic regression, management with BB was significantly associated with failure to gain PFC (OR 0.24; 95% CI 0.08–0.74), as well as increased hospital mortality (OR 3.81; 95% CI 1.25–11.57). Patients with PSC conversely were significantly more likely to attain PFC (OR 4.14; 95% CI 1.25–13.69) and less likely to die while hospitalized (OR 0.23; 95% CI 0.07–0.74) (Tables 3 and 4).

**Table 3:** Odds ratios (ORs) and associated 95% confidence intervals (CIs) for the association between initial abdominal closure technique and complications

	*N* (%)	Odds ratio	95% confidence interval	
			Lower	Upper
Fascial closure				
Skin only	133 (96.4)	4.14	1.25	13.69
ABThera	34 (94.4)	1.52	0.31	7.32
Bogotá bag	50 (83.3)	0.24	0.08	0.74
Barker’s vacuum packing	4 (80.0)	0.26	0.02	2.73
Hospital mortality				
Skin only	5 (3.6)	0.23	0.07	0.74
ABThera	5 (13.9)	1.48	0.45	4.85
Bogotá bag	9 (15.0)	3.81	1.25	11.57
Barker’s vacuum packing	0 (0)	1.00	–	–
Repeat bleeding				
Skin only	7 (5.1)	0.55	0.19	1.59
ABThera	2 (5.6)	0.39	0.08	1.87
Bogotá bag	8 (13.3)	1.96	0.69	5.55
Barker’s vacuum packing	2 (40.0)	18.32	2.29	146.92

^*^Multivariate logistic regression adjusted for age, gender, Injury Severity Score, mechanism, and massive transfusion requirement (> 10 units RBC/24 h)

**Table 4:** Odds ratios (ORs) and associated 95% confidence intervals (CIs) for the association between initial abdominal closure technique and complications

	*N* (%)	Odds ratio	95% confidence interval	
			Lower	Upper
Fascial closure				
Skin only	133 (96.4)	3.38	1.08	10.55
ABThera	34 (94.4)	1.83	0.38	8.75
Bogotá bag	50 (83.3)	0.26	0.09	0.73
Barker’s vacuum packing	4 (80.0)	0.35	0.03	3.69

^*^Multivariate logistic regression adjusted for Injury Severity Score, admission lactate, admission base deficit, and massive transfusion requirement (> 10 units RBC/24 h)

## Discussion

Our objective was to evaluate outcomes following DCS based on the role of initial TAC, with the primary outcome of PFC during the index hospitalization and prevention of LOD. We identified that patients undergoing PSC were able to undergo PFC at significantly higher rates than patients managed with other methods of initial TAC on both univariate and multivariate analyses. These patients though had significantly lower admission lactate, base excess, and transfusion requirements when compared to patients in the ABT and BB cohorts, who were matched in terms of demographics and injury severity. We did not identify a difference in rates of PFC between ABT and BB. However, when adjusting for potential confounding covariates, BB was significantly associated with LOD as well as increased hospital mortality whereas ABT was associated with increased ability to achieve PFC and increased hospital mortality, although not significant.

Patients managed with PSC historically suffered from increased rates of abdominal compartment syndrome given the inability of the re-approximated skin to comply with increasing visceral edema [9, 30, 31]. Such patients suffered from greatly elevated rates of LOD and mortality, and PSC was largely abandoned in spite of the ease with which it may be performed. However within the present cohort, use of PSC was significantly more likely to result in PFC, despite adjustment for potential confounding variables. The improvement in our outcomes is likely twofold.

Patients managed with PSC suffered less injury burden, as demonstrated by the significantly lower levels of admission lactate, base deficit, and transfusion requirements. At our institution, the decision for DCS and the type of TAC is based on the clinical judgment of the operative surgeon. Nationally, there is wide variability in the frequency of and indication for use of DCS, and our institution is no different [32]. Given this, the improved outcomes may simply result from selection bias. However, PSC continued to demonstrate improved outcomes when adjusting for confounders such as ISS and massive transfusion requirement. With this in mind, additional factors must be responsible for our improvement in outcomes. It is likely that significant bowel edema had not developed in this patient group to preclude PSC as a means to close the abdomen. Over the last 15 years, resuscitation strategies have evolved to complement DCS. The focus of DCR places an emphasis on blood product over crystalloid in the treatment of hemorrhagic shock, thus limiting the severe edema previously seen with massive IV fluid resuscitation [33–35]. Thus, PSC may still be utilized with success in patients undergoing DCS. However, caution must be taken with this interpretation given the increased risk of complications with delayed abdominal closure [36].

Patients managed initially with BB demonstrated significantly worse outcomes, despite lower ISS, similar levels of admission lactate and base excess, and requirement for blood product transfusion compared with patients managed initially with ABT. The complications we identified associated with BB are likely inherent in its design, as LOD remains a problem with BB despite the same changes in resuscitation strategies that may allow PSC to be a viable option. Fixation of IV bags to the skin does not allow drainage of intra-abdominal fluid that develops during the resuscitation phase that is critical in DCS [13]. This is compounded by lateral retraction of the skin and fascial edges [37]. As seen in our study, the times to abdominal closure were longest in the cohort managed with BB.

Previous studies raise concern that negative pressure vacuum therapy may potentiate further bleeding and risk enteric injury [22, 38]. However, the results from our study oppose these findings. The outcome improvements in our population may potentially be related to development of systems like ABT, which allow for better distribution of negative pressure and more uniform drainage of effluent from the peritoneal cavity [39]. Decreased intra-abdominal fluid allows for decreased intra-abdominal pressure, while also maintaining continued fascial traction toward the midline, thus allowing for earlier abdominal closure. Earlier and improvised methods of negative pressure vacuum therapy rely on a centralized negative pressure source which is both uneven and may leave areas of the peritoneal cavity undrained.

Effective drainage of the peritoneal cavity during TAC may offer additional benefits outside of pressure-related effects. Recent studies have highlighted the profound role that the peritoneal cavity may play as an inflammatory reservoir [20, 40]. New techniques incorporating peritoneal resuscitation with dialysate in conjunction with existing negative pressure vacuum TAC have been reported with significant decreases in mortality, time to closure, and rates of PFC [41, 42]. While other methods for TAC have sought to improve outcomes by preventing fascial retraction using various devices to physically keep the fascia at midline, increasing basic and translational research suggests that the benefits of direct peritoneal resuscitation stem from drainage of inflammatory mediators and modulation of organ damage, while also better providing resuscitative fluids [43–47]. Additional research efforts should be directed into the role of peritoneal resuscitation and drainage given these promising early results, which include a single-center, randomized, prospective trial [41].

Our study is not without limitations. Given its retrospective nature and the inherent limits of our medical record, we were unable to assess patients for abdominal compartment syndrome, as there is no regular recording of intra-abdominal pressure. Additionally, variation in practice patterns may have influenced outcomes. Though primarily validated in the treatment of hemorrhagic shock and traumatic coagulopathy, enthusiasm for DCS has encouraged use of the procedure for re-visualization or delayed repair of hollow viscus injury. Coupled with the potential of surgeons to use one personally preferred method of TAC, this may have introduced selection bias into the different cohorts. Finally, more patients are required to adequately evaluate the use of BVP and conclusions cannot be drawn regarding its use given our limited sample size. In spite of these limitations, we provide a large examination of multiple methods for TAC. To our knowledge, this is among the largest series reported on use of PSC or BB for DCS in the era of DCR.

## Conclusions

Our findings suggest that PSC is a viable option in the initial management of the open abdomen. This recommendation must acknowledge that these patients demonstrated less injury burden in comparison to others within our study. Concern for overuse of DCS has previously been raised and fervor for the procedure should be tempered with the knowledge that the risk of complications for patients managed with TAC is greater for those who may undergo definitive closure at the initial operation [36, 48]. Use of vacuum-assisted dressings continues to be the preferred method for TAC in DCS for trauma.

## Abbreviation

ABT: ABThera™ VAC system
BB: Bogota bag
BVP: Barker’s vacuum-packing
DCR: Damage control resuscitation
DCS: Damage control surgery
LOD: Loss of abdominal domain
PFC: Primary fascial closure
PSC: Primary skin closure
TAC: Temporary abdominal closure
